# Effect of Fatiguing Wheelchair Propulsion and Weight Relief Lifts on Subacromial Space in Wheelchair Users

**DOI:** 10.3389/fresc.2022.849629

**Published:** 2022-04-27

**Authors:** Ursina Arnet, Michael L. Boninger, Ann Cools, Fransiska M. Bossuyt

**Affiliations:** ^1^Shoulder Health and Mobility Group, Swiss Paraplegic Research, Nottwil, Switzerland; ^2^Department of Physical Medicine and Rehabilitation, School of Medicine, University of Pittsburgh, Pittsburgh, PA, United States; ^3^Department of Rehabilitation Sciences and Physiotherapy, University of Ghent, Ghent, Belgium; ^4^Human Performance Laboratory, Faculty of Kinesiology, University of Calgary, Calgary, AB, Canada

**Keywords:** acromiohumeral distance, occupation ratio, subacromial pain syndrome, impingement, spinal cord injury, fatigue, rotator cuff, shoulder pain

## Abstract

**Objective:**

This study aimed to identify targets of intervention for reducing shoulder pain in wheelchair users with spinal cord injury (SCI) by (1) examining changes in subacromial space [acromiohumeral distance (AHD) and occupation ratio (OccRatio)] with fatiguing wheelchair propulsion, and different loading conditions [unloaded position vs. weight relief lifts (WRL)]; (2) associating these changes with wheelchair user capacity, as well as (3) identifying subject characteristics associated with subacromial space, such as sex, lesion level, time since injury, body mass index and impaired shoulder range of motion.

**Methods:**

Fifty manual wheelchair users with SCI [11 females, age = 50.5 (9.7) years, time since injury = 26.2 (11.4) years] participated in this quasi-experimental one-group pretest-posttest study. Ultrasound images were used to define AHD during an unloaded position, and during personal and instructed WRL before and after fatiguing wheelchair propulsion. Furthermore, supraspinatus and biceps thickness defined from ultrasound images were used to calculate OccRatios. Wheelchair user capacity was quantified as functional strength (maximum resultant force reached during maximum isometric forward push) and anaerobic work capacity (highest power output reached during 15-m sprint test). Multilevel mixed-effects linear regression analyses controlling for between subject variability and covariables were performed to address the research questions.

**Results:**

AHD was significantly smaller during personal WRL (*p* < 0.001) and instructed WRL (*p* = 0.009, AHD both 11.5 mm) compared to the unloaded position (11.9 mm). A higher wheelchair user capacity (higher anaerobic work capacity) reduced the impact of WRL on AHD decrease. The fatiguing wheelchair propulsion had no effect on AHD (*p* = 0.570) and on OccRatio of supraspinatus (*p* = 0.404) and biceps (*p* = 0.448). Subject characteristics related to a larger subacromial space were lower lesion level, shorter time since injury, impaired external rotation, a lower body mass index and a higher anaerobic work capacity.

**Conclusion:**

This study showed a significant reduction in AHD during WRL with no effect of fatiguing wheelchair propulsion on the subacromial space in wheelchair users with SCI. A higher anaerobic work capacity was beneficial in stabilizing the shoulder during WRL. Our findings may assist clinicians in designing a shoulder injury prevention program.

## Introduction

Wheelchair users with spinal cord injury (SCI) face high demands on the upper extremity during ambulation, transfers, weight relief lifts (WRL) and numerous other activities of daily living. Especially the shoulder is at high risk for injury and pain. A recent review study reported a pooled prevalence of 44% of shoulder pain in wheelchair users (1).

Pathologies of the rotator cuff have been recognized as one of the main causes of shoulder pain in a general population (2). In manual wheelchair users, rotator cuff disorders are highly present, with supraspinatus tendons most often affected (84-100%) (3, 4). Also, pathologies of the biceps tendons are commonly detected (67-80%) (3, 4). Both tendons pass through the subacromial space and might be compressed due to narrowing of the available space between the humerus and the coracoacromial arch of the scapula. This may result in inflammation, chronic tendon degeneration and/or tendon rupture. Thus, narrowing of the subacromial space is hypothesized as one possible extrinsic mechanism that contributes to shoulder pain (5). Acromiohumeral distance (AHD), which is the shortest linear distance between the most inferior aspect of the acromion and the adjacent humeral head, is a good indicator of the size of the subacromial space and has previously been used to quantify the risk for subacromial pain syndrome (6). The occupation ratio (OccRatio) is defined as the percentage of AHD that is occupied by the tendon (7). This ratio might be even more informative regarding risk for subacromial pain syndrome than absolute distance, since tendon thickness can also change. Both parameters, AHD and OccRatio, can be measured by ultrasound with reliable and consistent results. For AHD, good to excellent intraclass correlation coefficients (ICC) of 0.85-0.98 for intra-rater reliability and 0.88-0.94 for inter-rater reliability were reported (8–10). Regarding OccRatio, ICC values of 0.88-0.92 for intra-rater reliability and 0.79 for inter-rater reliability were reported by BaGcier et al. (8).

The daily demands on the wheelchair users' shoulder may influence OccRatio and therefore the risk for shoulder complaints. The high load acting on the shoulder during weight lifting tasks, such as transfers or WRL for pressure injury prevention might reduce AHD due to cranial humerus migration into the subacromial space (11). The movement of the scapula with respect to the humeral head might further reduce the available subacromial space during these tasks (11). Furthering the risk, the repetitiveness of wheelchair propulsion might fatigue the rotator cuff muscles and change their tendon properties (12) as well as their capability to stabilize the shoulder joint. A better wheelchair user capacity, e. g. higher anaerobic work capacity or functional shoulder muscle strength, might enable a better shoulder stabilization and thus reduce the risk for subacromial pain syndrome (13).

With this study we aimed to identify targets of intervention for reducing shoulder pain in wheelchair users with SCI. The goal of the study was (1) to examine changes in subacromial space (AHD and OccRatio) with fatigue due to wheelchair propulsion, and different loading conditions (unloaded position vs. WRL); (2) to associate these changes with wheelchair user capacity (functional strength and anaerobic work capacity), as well as (3) to identify subject characteristics associated with subacromial space, such as sex, lesion level, time since injury, body mass index (BMI) and impaired shoulder range of motion (ROM). We hypothesized that there will be a significant decrease in AHD and OccRatio due to fatiguing wheelchair propulsion and different loading conditions, and that greater changes will be observed in wheelchair users with a lower capacity.

## Materials and Methods

### Study Design and Participants

The study has a quasi-experimental one-group pretest-posttest design (ClinicalTrials.gov, identifier: NCT03153033). Parts of the data collected for this study were published elsewhere (12, 14).

A sample of 50 participants was recruited from the population-based Swiss Spinal Cord Injury Cohort study (SwiSCI) database (15). Inclusion criteria of the study were (1) nonprogressive traumatic or non-traumatic SCI, (2) diagnosed neurological lesion level at T2 or below, (3) at least 1 year post discharge from rehabilitation, (4) between 18 and 65 years old, (5) daily use of a pushrim wheelchair and no required support for propelling for more than 100 m, and (6) quick-release axle to remove wheels from the wheelchair in order to attach a measurement wheel during the later experiment. Exclusion criteria were (1) receiving palliative care, (2) SCI due to congenital conditions, persons with neurodegenerative disorders, or Guillain–Barré syndrome, (3) upper-extremity pain that limits the ability to propel a wheelchair, (4) history of shoulder, elbow, or wrist fractures/dislocations that are still causing symptoms, and (5) history of cardiopulmonary problems that could be exacerbated by strenuous physical activity. In a first step, eligible participants were selected in the SwiSCI database fulfilling inclusion criteria 1, 2, 3, and 4. Subsequently, an information letter including a description of the study, all intended measurements and requirements, as well as a short questionnaire to verify the remaining inclusion and exclusion criteria was sent to the eligible participants. With this procedure a sample size of 50 participants was reached.

Prior to data collection, ethical approval was obtained from the Ethikkommision Nordwest-und Zentralschweiz and all participants read and signed the informed consent.

### Procedure

Participants were invited for one testing session at the biomechanical laboratory of Swiss Paraplegic Research. They were instructed to avoid strenuous exercises 48 h prior to testing.

**Figure 1:**
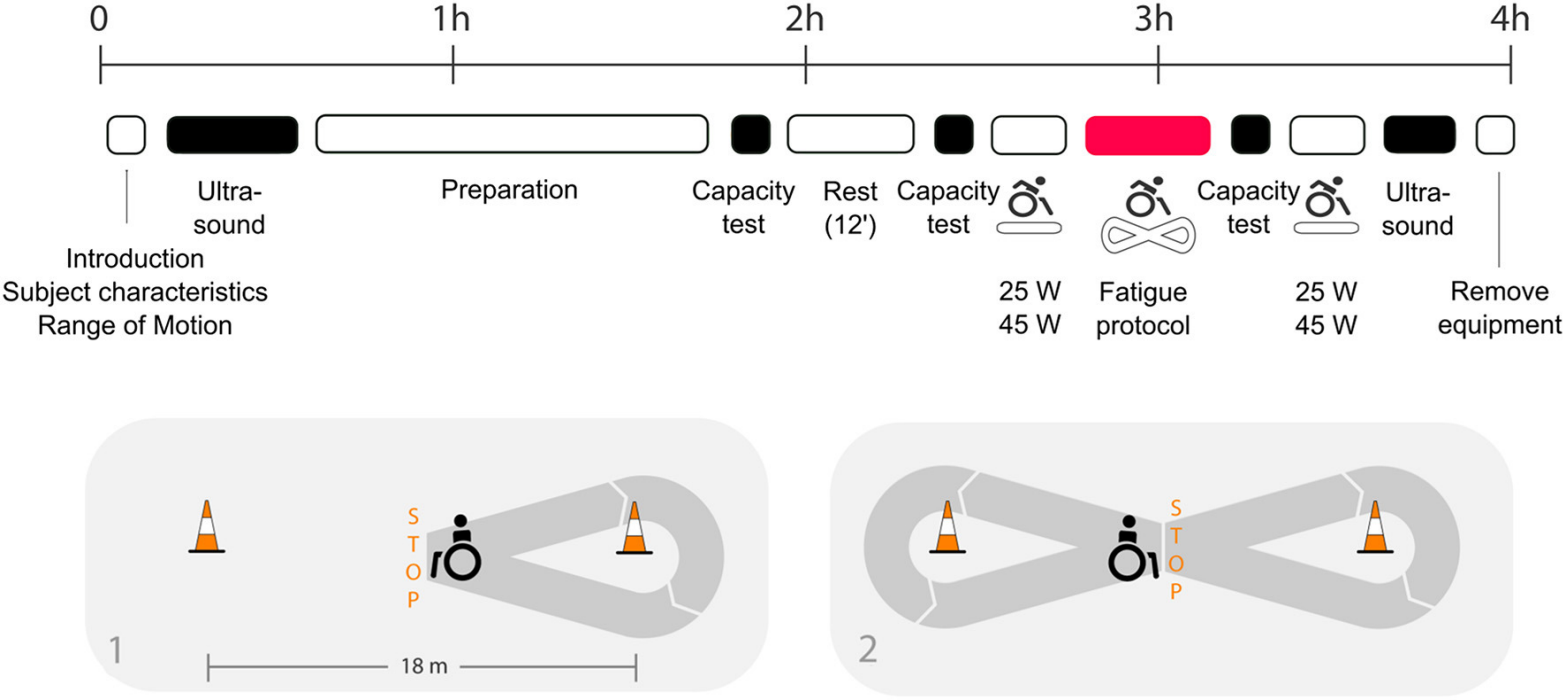
Figure adjusted from (12): timeline of the assessments taken in the biomechanical laboratory including (1) introduction, self-reported subject characteristics and measurements of the shoulder range of motion, (2) ultrasound exams (pre and post fatigue), (3) preparation phase including a test to define individual drag force and familiarization with treadmill propulsion and fatigue protocol, (4) wheelchair user capacity test (capacity test): three maximum push tests and a maximum 15 m overground sprint test (pre and post fatigue), (5) passive rest phase, (6) manual wheelchair (MWC) propulsion at two different conditions (25 and 45 W, pre and post fatigue), and (7) fatigue protocol: overground wheelchair propulsion along an eight-shaped course. The detailed course of the fatigue protocol is presented below the timeline.

Several measurements were conducted before and after standardized wheelchair propulsion on a treadmill and a fatiguing intervention of 15 min. The fatiguing intervention was a figure-8 protocol, consisting of three 4-min intervals of maximum voluntary wheelchair propulsion including right and left turns, start and stops, separated by 90 s of rest (total duration of 15 min, Figure 1). For that, two cones were placed 18 m apart on a concrete floor and the participants started in the middle of the cones. They were instructed to propel after the start signal as fast as possible toward the first cone, make a right turn around the cone and stop at the starting point. Immediately after a full stop they propelled with a left turn at maximum speed around the second cone and stopped again in at the starting point. This figure-8 was repeated as often as possible within 4 min. Instructions given during fatiguing interventions were standardized. The protocol has been used before in combination with ultrasound examinations (16).

### Data Collection and Analysis

#### Subject Characteristics

After introduction of the study and signing the informed consent, participants were asked to self-report socio-demographic variables (age, sex, and height), characteristics of the injury (traumatic or non-traumatic etiology, date of injury, completeness of the injury, and neurological lesion level). Weight was collected with a wheelchair scale by subtracting the weight of the wheelchair from the total weight.

#### Range of Motion

Passive shoulder range of motion was measured prior to the fatiguing intervention with a goniometer while sitting in the wheelchair. Shoulder range of motion was classified as impaired when meeting the following criteria: anteroflexion < 170 °, external rotation < 50° or abduction < 170°.

#### Wheelchair User Capacity

The wheelchair user capacity tests were performed prior and after the fatiguing intervention. Wheelchair user capacity tests consisted of functional strength test and anaerobic work capacity tests. During the capacity tests, 3-dimensional forces and moments applied to the pushrim were collected at 240 Hz with the SmartWheel (Three Rivers Holdings, Inc, Mesa, AZ) fitted to the non-dominant side of the participants' personal wheelchair. The non-dominant side was chosen as this project aims to investigate the shoulder most predominantly affected by wheelchair propulsion and less by other activities of daily living, such as overhead reaching, lifting objects, etc. A dummy wheel with an equal tire as the SmartWheel was attached to the contralateral side.

To evaluate functional strength, participants performed three times a 5-s maximum isometric forward push with hands on top of the pushrim and wheelchair attached from behind to restrict forward movement (17). Functional strength was defined as the maximum resultant force reached during the three maximum isometric forward pushes.

To determine anaerobic work capacity a 15 meter overground wheelchair sprint was completed prior to the fatiguing intervention (17). The outcome was the peak power output measured during the sprint test.

#### Ultrasound: AHD, Tendon Thickness and Occupation Ratio

Ultrasound images of the supraspinatus tendon and the subacromial space of the non-dominant shoulder were taken before any propulsion activity and following the fatiguing intervention. A single examiner (FMB) took all ultrasound images in a randomized order (NextGen Logiq TM e R90.2, GE Healthcare, USA). Image field depth was set at 4 cm and gain was set at 60 dB. To allow for repeated measurements before and after the propulsion tasks with limited error in variation of probe location, a steel reference marker was taped to the skin.

**Figure 2:**
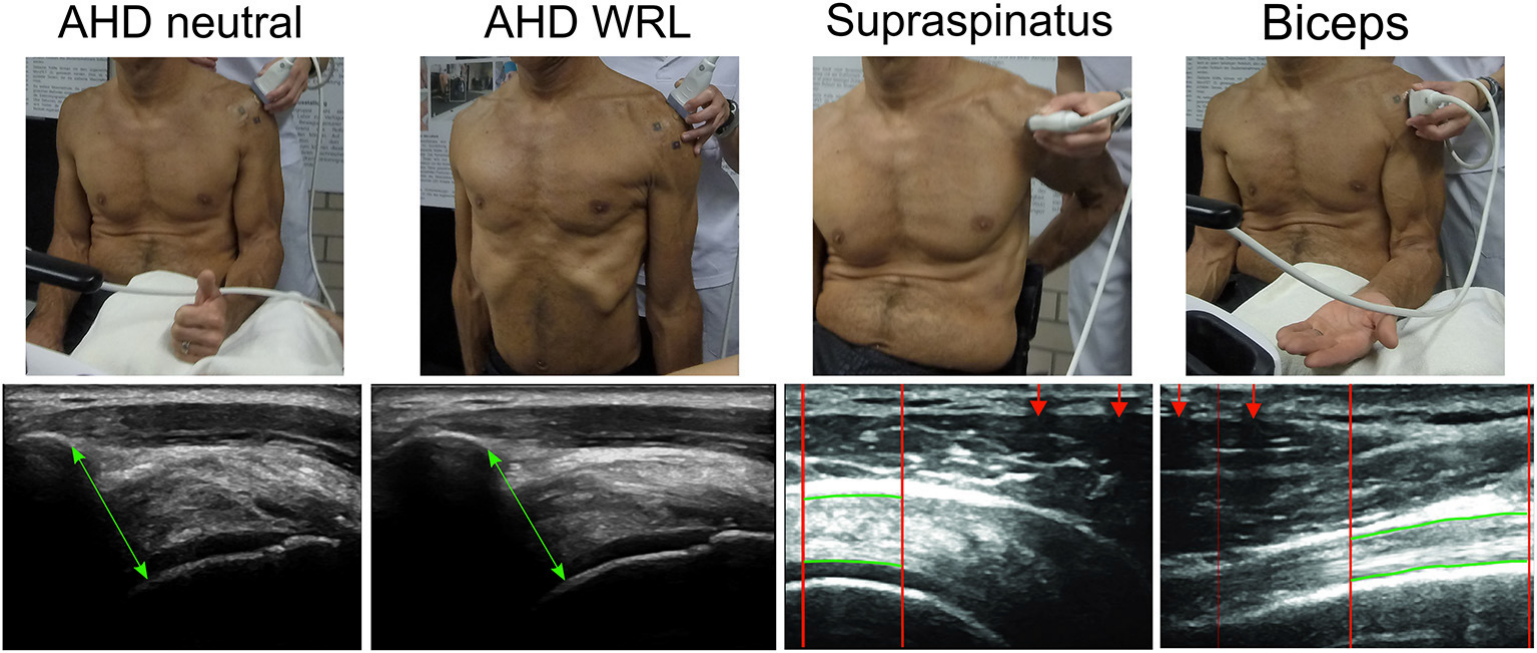
Ultrasound measurements: position and example of measurement. Each type of measurements represents a different example participant and does not relate to the person of the position images. AHD neutral: acromiohumeral distance images in the neutral position were taken in a seated position with 90° elbow flexion with the thumb facing upward. AHD WRL: acromiohumeral distance images during WRL. Supraspinatus: transverse ultrasound images of supraspinatus tendon were taken in a seated position with the palm placed on the lower back, shoulder extended, and the elbow flexed posteriorly. Biceps: longitudinal images of long head of biceps brachii tendon were taken in a seated position with 90° elbow flexion and the hand palm facing upward. The region of interest (ROI) is presented between the red vertical lines with the upper border and lower border of the tendons marked with the manually identified red horizontal lines. Lines are marked thicker as compared with the actual analysis for visualization reasons. ROI is selected based on the interference pattern that resulted from a metal marker taped to the skin (assigned with red arrows).

For quantifying AHD in an unloaded position, three images were taken during 90° elbow flexion with the thumb facing upward (Figure 2). Furthermore, three images of the AHD were taken during WRL without any instructions given (personal WRL), and during instructed WRL, where participants were asked to depress and retract the shoulders (Figure 2). AHD was defined as the shortest distance between the anterior inferior edge of the acromion and the most superior aspect of the humerus (10). The average measure of the three repeated measures was always used. All blinded ultrasound images were analyzed in randomized order by a single examiner (FMB) using Matlab R2016b custom programs (Mathworks, Inc., Natick, MA, USA).

For quantifying supraspinatus tendon thickness, two transverse images were taken in a seated position with the palm placed on the lower back, the shoulder extended, and the elbow flexed posteriorly (Figure 2). For the tendon of the long head of the biceps brachii, two longitudinal images were taken in a seated position with 90° elbow flexion and the hand palm facing upward while resting on a cushion (Figure 2). The region of interest of each ultrasound image was defined from the interference pattern at the top of the images, created from the steel reference markers attached to the skin. Within the region of interest, tendon thickness was measured as the mean distance between top and bottom border of the tendon and the average of the two repeated measures was used.

The occupation ratio expresses the tendon thickness relative to the available subacromial space. Occupation ratio of the supraspinatus and biceps tendon was calculated as the percentage of the mean tendon thickness relative to the mean AHD (7). Occupation ratio was only calculated for the unloaded position since tendon thickness was not measured during WRL.

### Statistical Analysis

Statistical analyses were conducted with STATA software (version 16.1, StatCorp, LP, College Station TX, USA). Multilevel mixed-effects linear regression analyses controlling for between subject variability and covariables were performed to:

1) identify the association between the dependent variable AHD and different loading conditions (neural position, personal WRL and instructed WRL) before and after fatigue. Interactions between wheelchair user capacity (functional strength, anaerobic work capacity) with time and loading conditions were included.

2) identify the association between the dependent variable OccRatio of the supraspinatus and biceps tendon before and after fatigue, including interactions with wheelchair user capacity.

Covariables included known risk factors for shoulder pain, such as subject characteristics (sex, lesion level, body mass index (kg/m^2^), years since injury), impaired shoulder range of motion [in anteroflexion (<170°), external rotation (<50°), and abduction (<170°)], and wheelchair user capacity. If a significant difference (α = 0.05) was found between time points or loading conditions, pairwise comparisons with Bonferroni corrections were used to evaluate differences.

## Results

Subject and lesion characteristics of the 50 participants [mean age 50.5 (SD 9.7) years, 11 females, 39 males] are listed in Table 1. Mean time since injury was 26.2 (SD 11.4) years and the majority of the participants had a complete lesion (78%). Range of motion in anteroflexion was most often impaired (in 84% of participants), followed by abduction (48%) and external rotation (24%). Regarding wheelchair user capacity, a mean functional strength of 221 N (SD 49 N) was reached during the isometric forward push and a mean power output of 84 W (SD 32 W) was measured during the sprint test.

**Table 1:** Subject characteristics, lesion characteristics, and wheelchair user capacity [% or mean (SD)] for the total sample and stratified by lesion level.

		**Lesion level**		
	**Total**	**T2-T6**	**T7-T12**	**L1-L2**
	**(*n* = 50)**	**(*n* = 20)**	**(*n* = 22)**	**(*n* = 8)**
Sex (% male)	78	95	68	63
Age (years)	50.5 (9.7)	48.4 (10.4)	50.5 (9.6)	56.0 (6.7)
Weight (kg)	72.4 (13.3)	73.4 (12.6)	69.6 (13.0)	77.4 (15.7)
BMI (kg/m^2^)	24.0 (4.4)	23.6 (4.1)	23.1 (3.7)	27.5 (5.4)
Time since injury (years)	26.2 (11.4)	27.2 (11.3)	24.9 (11.4)	27.3 (13.1)
Lesion completeness (% complete)	78	90	77	50
ROM anteroflexion (% impaired)	84	100	73	75
ROM abduction (% impaired)	48	55	46	38
ROM exorotation (% impaired)	24	30	27	0
FrMaxpush (N)	221 (49)	229 (43)	214 (54)	222 (51)
Sprint peak power output (W)	84 (32)	76 (26)	91 (37)	83 (31)

*BMI, body mass index; ROM, range of motion; FrMaxpush, maximum resultant force reached during the three maximum isometric forward pushes*.

### Acromiohumeral Distance

AHD values measured pre- and post-fatigue, as well as during different positions (neutral, personal WRL, and instructed WRL) can be found in Table 2. AHD was smaller during WRLs compared to the unloaded position. When controlling for all covariables, AHD was significantly larger during the unloaded position [mean 11.9 mm, 95% confidence interval (CI) 11.3-12.5 mm] compared to the personal WRL (mean 11.5 mm, CI 10.9-12.1 mm, *p* < 0.001) and instructed WRL (mean 11.5 mm, CI 10.9-12.1 mm, *p* = 0.009).

**Table 2:** Unadjusted values [mean (SD)] of the dependent variables acromio-humeral distance (AHD) and occupation ratio (OccRatio) of supraspinatus and biceps tendon pre- and post-fatiguing wheelchair propulsion and during different positions: neutral, personal weight relief (pWRL) and instructed weight relief (iWRL).

**Time**		**Pre**			**Post**			**Mixed Model p values**	
**Position**	***n***	**Neutral**	**pWRL**	**iWRL**	**Neutral**	**pWRL**	**iWRL**	**time**	**Position**
AHD (mm)	50	11.8 (2.8)	11.5 (2.5)	11.5 (2.6)	12.0 (3.0)	11.6 (2.7)	11.6 (2.7)	0.570	<0.001^a^, 0.009^b^, 0.112^c^ ♦
OccRatio supraspinatus (%)	50	47.3 (12.2)			45.7 (14.5)			0.404	
OccRatio biceps (%)	50	37.5 (17.9)			37.2 (15.2)			0.448	

*Pairwise comparisons: ^a^neutral vs. pWRL, ^b^neutral vs. iWRL, ^c^pWRL vs. iWRL*.

*Significant interactions (alpha = 0.05) from the mixed-effects multilevel analysis: ♦ = interaction position x capacity (sprint)*.

No effect of the fatiguing wheelchair propulsion on AHD was found (Table 2). When controlling for all covariables, AHD pre-fatigue (mean 11.6 mm, CI 11.0-12.2 mm) was not significantly different than AHD post-fatigue (mean 11.7 mm, CI 11.1-12.3 mm, *p* = 0.570).

**Figure 3:**
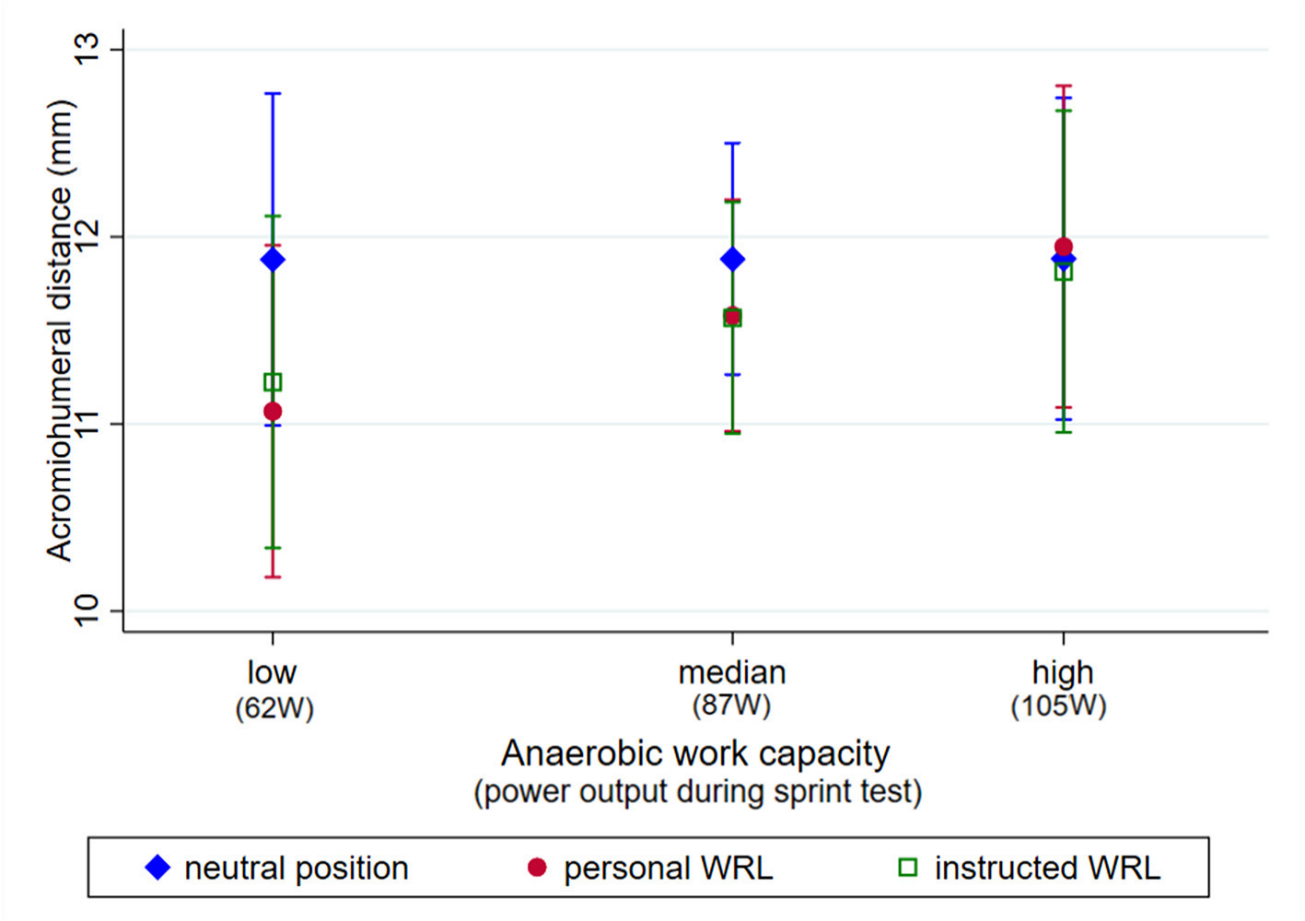
Predictive margins with 95% confidence interval of acromiohumeral distance (AHD, mm) measured during different loading positions (unloaded position, personal WRL, and instructed WRL) in participants with a low (62 W, 25% percentile), median (87%) and high (105, 25% percentile) anaerobic work capacity.

There was a significant interaction effect of position and anaerobic work capacity (*p* < 0.001). Participants who reached a lower power output during the sprint test (low anaerobic work capacity) had reduced AHDs during the WRLs compared to the unloaded position. In participants with a higher anaerobic work capacity there was no difference in AHD between unloaded position and WRLs (Figure 3).

**Figure 4:**
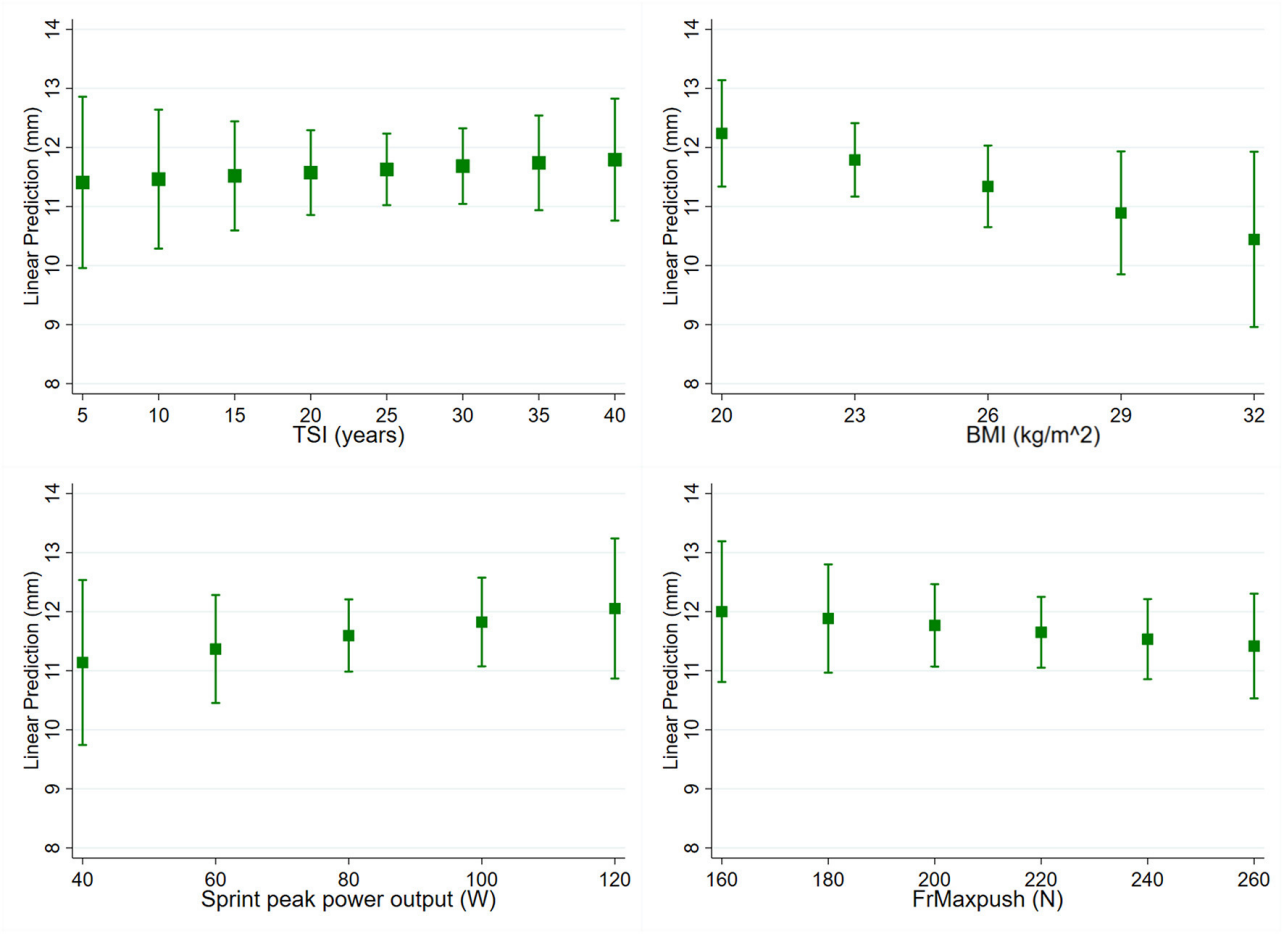
Predictive margins with 95% confidence interval of acromiohumeral distance (AHD, mm) for continuous covariables time since injury [(TSI), *p* = 0.727], body mass index [(BMI), *p* = 0.083], sprint peak power output (*p* = 0.941) and resultant force reached during maximum isometric forward pushes [(FrMaxpush), *p* = 0.418].

There were significant associations of AHD and lesion level, as well as impaired ROM (Table 3). Participants with lower lesion levels (L1-L2) had a significantly larger AHD (mean 15.2 mm, CI 13.5-16.8mm) than participants with higher lesion levels (T7-T12: mean 11.0 mm, CI 10.0-12.1 mm, T2-T6: mean 10.9 mm, CI 9.7-12.0 mm, both *p* < 0.001). Participants with an impaired ROM in external rotation (<50°) had a larger mean AHD of 13.2 mm (CI 11.9-14.9 mm) compared to participants with no impairments of external rotation ROM (mean 11.1 mm, CI 10.4-11.8, *p* = 0.008). There were no significant associations with any other included subject characteristics (Table 3; Figure 4).

**Table 3:** Predictive margins with 95% confidence interval (95% CI) of acromiohumeral distance (AHD) and occupation ratio (OccRatio) of supraspinatus and biceps for categorical covariables sex, lesion level, shoulder range of motion in anteroflexion (AF), external rotation (ER) and abduction (ABD): predictive margins with 95% confidence intervals.

		**AHD**			**OccRatio supraspinatus**			**OccRatio biceps**		
		**Mean**	**95% CI**	***P***	**Mean**	**95% CI**	***P***	**Mean**	**95% CI**	***P***
Sex	Female	10.7	8.9-12.5	0.257	49.3	40.9-57.6	0.626	34.9	25.0-44.8	0.527
	Male	11.9	11.2-12.7		46.7	43.1-50.4		38.8	34.5-43.2	
Lesion	T2-T6	10.9	9.7-12.0	1.000^a^	48.4	43.0-53.8	1.000^a^	39.9	33.5-46.3	1.000^a^
level	T7-T12	11.0	10.0-12.1	<0.001^b^	49.7	44.7-54.6	0.127^b^	39.6	33.7-45.4	0.192^b^
	L1-L2	15.2	13.5-16.8	<0.001^c^	38.1	30.1-46.1	0.057^c^	28.8	19.3-38.3	0.192^c^
ROM	<170°	11.6	10.9-12.3	0.885	48.1	44.8-51.4	0.365	36.7	26.0-47.3	0.811
AF	>170°	11.8	9.9-13.7		43.4	34.4-52.3		38.1	34.3-42.0	
ROM	<170°	11.7	10.7-12.6	0.981	44.9	40.4-49.4	0.174	38.5	33.6-43.5	0.747
ABD	>170°	11.6	10.8-12.5		49.4	45.2-53.6		37.2	32.0-42.5	
ROM	<50°	13.2	11.9-14.9	0.008	39.5	46.5-53.2	0.005	30.2	23.0-37.5	0.019
ER	>50°	11.1	10.4-11.8		49.8	46.5-53.2		40.5	36.5-44.4	

*Pairwise comparisons: ^a^T2-T6 vs. T7-T12, ^b^T2-T6 vs. L1-L2, ^c^T7-T12 vs. L1-L2*.

### Occupation Ratio

OccRatios of the supraspinatus and biceps measured pre- and post-fatigue can be found in Table 2. The fatiguing wheelchair propulsion had no effect on the OccRatio of the supraspinatus and biceps. When controlling for all covariables, OccRatio of the supraspinatus was not significantly different pre-fatigue (mean 48.1%, CI 45.2-51.0%) compared to post-fatigue (mean 46.5%, CI 43.6-49.0%, *p* = 0.404). The same accounts for the OccRatio of the biceps, where mean pre-fatigue values of 38% (CI 34.6-41.5) and post-fatigue values of 37.8% (CI 34.4-41.2% *p* = 0.448) were found.

**Figure 5:**
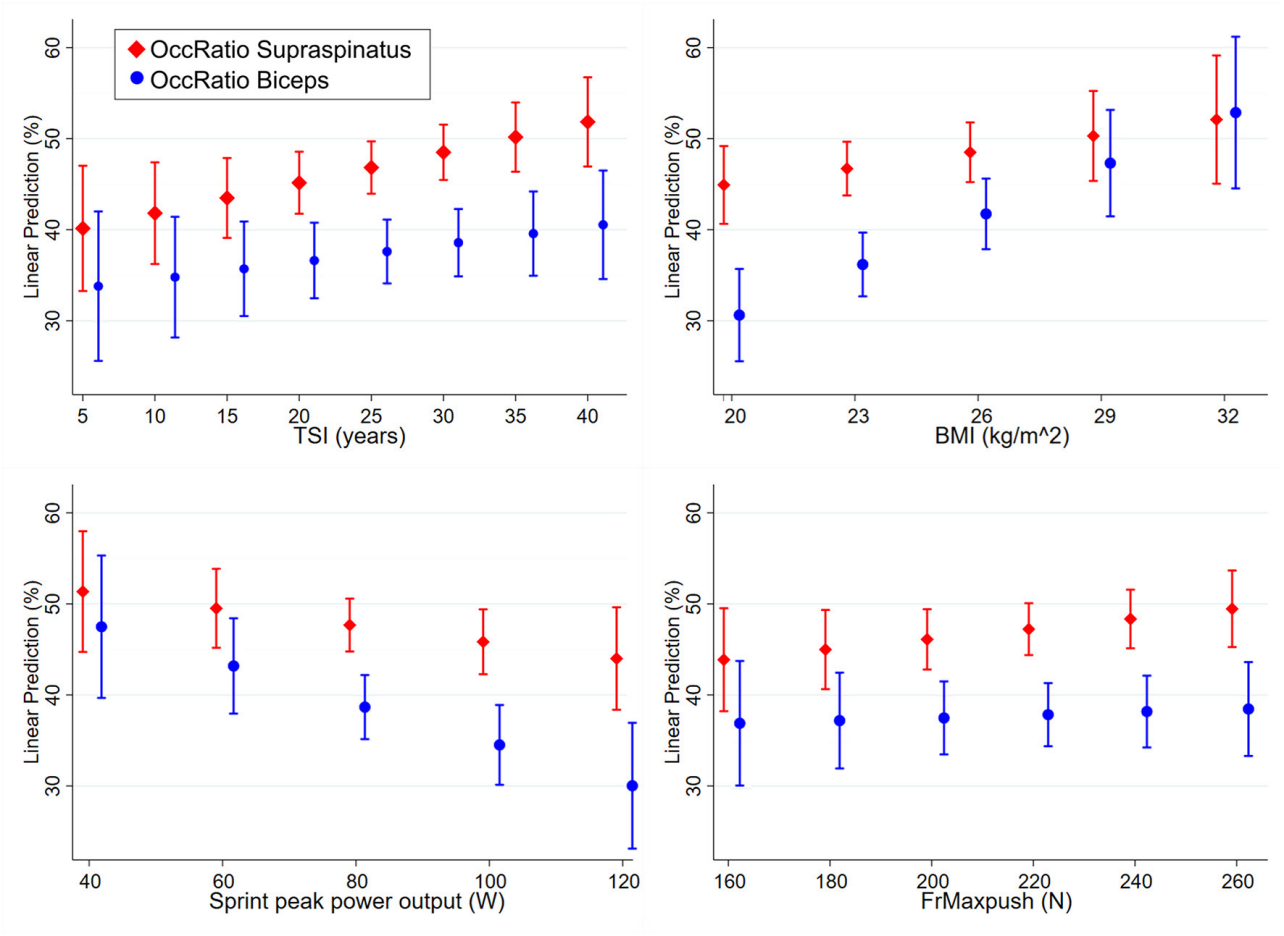
Predictive margins with 95% confidence interval of occupation ratio (OccRatio, %) of supraspinatus and biceps for continuous covariables time since injury [(TSI), supraspinatus *p* = 0.025, biceps *p* = 0.280], body mass index [(BMI), supraspinatus *p* = 0.144, biceps *p* < 0.001], sprint peak power output (supraspinatus *p* = 0.288, biceps *p* = 0.006) and resultant force reached during maximum isometric forward pushes [(FrMaxpush), supraspinatus *p* = 0.251, biceps *p* = 0.637).

Regarding supraspinatus, participants with a shorter time since injury had lower OccRatio (*p* = 0.025, Figure 5). When external rotation ROM was impaired (<50°), participants had a lower supraspinatus OccRatio (mean 39.5%, CI 46.5-53.2%) compared to unimpaired ROM (mean 49.8%, CI 46.5-53.2%, *p* = 0.005, Table 3). There were no other significant associations of supraspinatus OccRatio with the analyzed subject characteristics (Table 3; Figure 5).

OccRatio of the biceps was significantly smaller in participants with a lower BMI (*p* < 0.001, Figure 5), as well as in participants with a higher sprint peak power output (*p* = 0.006, Figure 5). When external rotation ROM was impaired (<50°), participants had a lower biceps OccRatio (mean 30.2%, CI 23.0-37.5%) compared to unimpaired ROM (mean 40.5%, CI 36.5-44.4% *p* = 0.019, Table 3). No other significant associations were found for biceps OccRatio (Table 3; Figure 5).

## Discussion

This study found a significant reduction in AHD during WRL compared to the unloaded position in 50 wheelchair users with SCI. This was mainly found in participants who reached a lower power output during the sprint test (low anaerobic work capacity). However, fatiguing wheelchair propulsion had no effect on subacromial space since neither AHD nor OccRatio of the supraspinatus and biceps tendon were changed after this intervention. Subject characteristics associated with a larger subacromial space were: lower lesion levels, shorter time since injury, a lower BMI, impaired external rotation ROM and a higher anaerobic work capacity.

### Subacromial Space and Associated Factors

AHD is considered as a good indicator of the size of the total subacromial space (9). In the studied population of wheelchair users with SCI, we found a mean AHD of 11.8 mm during the unloaded position, when the elbow was 90° flexed and the lower arm was supported. These values are slightly higher than previously reported values of 9.4 mm (18) to ~11 mm (9) in the same population of wheelchair users with SCI. Subacromial space quantified by AHD is an external factor that has been commonly investigated in patients with subacromial pain syndrome. However, no clear association between AHD values in resting position and subacromial pain syndrome was found in previous studies (6, 7, 19, 20).

OccRatio gives more detailed information on the available subacromial space than AHD by taking tendon thickness into account. When analyzing the subacromial space in relation to the space occupied by the tendons, we found a mean OccRatio of 47.3% for supraspinatus and 37.5% for biceps. These values are higher than previously reported OccRatios of 36.5% (supraspinatus) and 23.1% (biceps) in wheelchair users with SCI (21), but lower than OccRatio of the supraspinatus reported in asymptomatic able bodied individuals [53.5% (22), 56.4% (20)]. A lower OccRatio is seen as beneficial since less space is occupied by the tendon and more space is potentially available. In this line, higher OccRatios have been found in previous studies in persons with subacromial pain syndrome (7, 20, 22). These findings suggest that tendon thickness in relation to AHD should be considered when analyzing the risk for subacromial pain syndrome.

The present study found several subject characteristics associated to the size of the subacromial space. Participants with a shorter time since injury had a lower supraspinatus OccRatio. This indicates that with longer time in the wheelchair, and with more cumulated load on the shoulder, either supraspinatus tendon might increase or AHD decreases. Since AHD was not associated with time since injury in the present study, this change may be related to an adaption of the supraspinatus tendon over time as a response to chronic overload. This statement is supported by findings of Malanga et al. who found thicker supraspinatus tendons on the dominant side of baseball pitchers when comparing to the non-dominant side (23). Clinical practice guidelines recommend selective strengthening and stretching exercises for rotator cuff muscles in manual wheelchair users. The changes in the OccRatio further support this recommendation as such exercises may prevent pathology in the rotator cuff.

Participants with an impaired external rotation ROM had a lower OccRatio of supraspinatus and biceps, as well as a larger AHD. This finding points toward a mediating effect of the external rotators. Leong et al. (24) also reported a mediating effect of the external rotators since individuals with greater strength in external rotation presented larger AHD. Whether these findings are related or how muscular imbalance affects the subacromial space should be examined in future studies.

Finally, a lower BMI and a higher anaerobic work capacity was associated with a smaller biceps OccRatio. This supports the general recommendation that a reduced body weight and higher capacity is beneficial for the weight bearing shoulder (25). The effect of training has been evaluated in a previous study where individuals with subacromial pain syndrome participated in a rehabilitation program including strengthening of the rotator cuff and trunk muscles and endurance training. Savoie et al. found a significantly increased AHD after the rehabilitation program (13). This is a further indication that increasing wheelchair user capacity through training reduces the risk for subacromial pain syndrome.

### Effect of WRL on Subacromial Space

A temporary narrowing of the subacromial space due to high load or due to movement patterns of the shoulder structures is generally seen as a risk factor for compression of the soft tissue under the acromioclavicular arch and inflammation (26). Previous kinematic studies on the orientation of the scapula and humerus identified WRL as an activity of daily life of wheelchair users, where the risk for narrowing of the subacromial space is high (11, 27, 28). During a WRL, glenohumeral external rotation is decreased and the scapula is anteriorly tilted and internally rotated. This reduces the subacromial space, and in combination with the large superior forces at the shoulder (29), places the shoulder of the wheelchair user at high risk for compression of the structures in the subacromial space (11).

The present study found significantly decreased AHD during WRL, which indeed points to a risk for shoulder injury. Whether participants performed WRL in their own style (no instruction given) or whether they followed the instructions to ensure optimal shoulder position (depressed and retracted the shoulders) did not result in a significant difference in AHD. Similar reductions of AHD were found in previous studies (26). This reduction in AHD during WRL strengthens the current notion to avoid weight relief maneuvers that place high, superiorly directed forces on the arm. Whenever possible, alternative techniques for pressure relief like forward or side leans should be used (25).

A remarkable interaction effect was found for position (unloaded vs. WRL) and anaerobic work capacity. Participants with a low anaerobic work capacity (lower power output reached during the sprint test) presented the above-mentioned reduction in AHD between unloaded position vs. WRL. Participants with a higher anaerobic work capacity, however, could maintain their AHD also during WRL. These results highlight the importance of anaerobic work capacity in shoulder function in the context of WRL. A well-planned preventive training program that safely increases wheelchair user capacity may reduce shoulder complaints (30).

To our knowledge, no study analyzed OccRatio during WRL. Mozingo et al. took however tendon thickness of infraspinatus, subscapularis and supraspinatus into account and defined risk scores based on fluoroscopy images to estimate mechanical impingement risk (5). Their results showed only minimal to no impingement risk during pressure relief lifts. Despite these findings, the authors advised wheelchair users to perform side leans for pressure relief and pressure injury prevention instead of WRL to reduce loading of the shoulder.

### Effect of Fatigue on Subacromial Space

Fatigue of the muscles stabilizing the shoulder joint may reduce the subacromial space and increase stress on the tendons within the space. There are two fatigue-based mechanisms proposed to cause narrowing of the subacromial space: superior migration of the humeral head with respect to the glenoid and alteration of the movement of the acromion with respect to the humeral head due to fatigue (31). A simulation study including empirically generated fatigue data has shown that the subacromial space was affected by fatigue and that superior humeral migration was the dominant fatigue-related mechanism associated with shoulder injury risk (31).

Our intervention study, however, did not show an effect of fatigue on either AHD nor OccRatio. Also, no interaction with wheelchair user capacity was found. This suggests that daily wheelchair propulsion, as simulated in this study by the fatiguing intervention, does not contribute to temporary changes in subacromial space. Similar findings have been reported by Lin et al. who found in general no changes in subacromial space after performing repetitive WRL and shoulder external rotations (26). Participants with greater levels of shoulder pain, however, showed a greater percentage narrowing of AHD. The present study excluded participants with upper-extremity pain that limits the ability to propel a wheelchair. This might be an explanation why no narrowing of the subacromial space was found.

### Study Limitations

The intervention of fatiguing wheelchair propulsion used in this study was chosen to simulate everyday load acting on the shoulder of a wheelchair user. The fatiguing protocol included maximum voluntary overground propulsion, starting, stopping and turning. Other demanding tasks for the shoulder, such as transfers, WRL and lifting heavy objects were not included. For future studies on the effect of fatigue resulting from everyday life activities, these additional tasks could be included in the fatigue protocol as long as they can be performed in a safe way. However, the used protocol is expected to be more demanding than everyday life activities since it requires maximum voluntary propulsion.

Regarding measures to quantify subacromial space it should be considered that ultrasound images only allow for two-dimensional measurements and that the measures used to calculate OccRatio in this study (AHD and tendon thickness) were taken from different ultrasound images and with different arm positions of the participants (Figure 2). This has been done similarly in previous studies quantifying OccRatio (7–9). Unfortunately, the arm position used to measure thickness of the supraspinatus makes it impossible to quantify this thickness and thus supraspinatus OccRatio during WRL. Since OccRatio is more informative on the available subacromial space and thus on the shoulder injury risk, the quantification of OccRatio during WRL would be an interesting venue for the future if technology and analysis software allow.

While we excluded individuals with upper-extremity pain that limits the ability to propel a wheelchair, participants may still have had pain. Future studies should look at the impact of pain on the measures collected in this study.

## Conclusions and Implications

This study showed a significant reduction of the AHD during WRL compared to the unloaded position in wheelchair users with SCI. A higher anaerobic work capacity reduced the impact of WRL on AHD decrease and was thus beneficial in stabilizing the shoulder. Fatiguing wheelchair propulsion had no effect on the subacromial space. Subject characteristics related to a larger subacromial space were lower lesion level, shorter time since injury, impaired ROM in external rotation, a lower BMI and a higher anaerobic work capacity. Preventive fitness training to increase wheelchair user capacity, alternative modes for pressure relief and lowering BMI are suggested interventions to lower the risk for subacromial pain syndrome in wheelchair users with SCI. These findings may assist clinicians in designing injury prevention programs.

## Data Availability Statement

The datasets generated and/or analyzed during the current study are available from the corresponding author on reasonable request.

## Ethics Statement

The studies involving human participants were reviewed and approved by Ethikkommision Nordwest-und Zentralschweiz, Switzerland. The patients/participants provided their written informed consent to participate in this study. Written informed consent was obtained from the individual(s) for the publication of any potentially identifiable images or data included in this article.

## Author Contributions

UA, FB, and MB initiated the study. UA, FB, MB, and AC contributed to the conception and design of the study. FB performed the data collection. UA was responsible for all analyses, drafting, and finalization of the paper. All authors critically revised the paper and have read and approved the final paper.

## Funding

Swiss Paraplegic Research: open access publication fee.

## Conflict of Interest

The authors declare that the research was conducted in the absence of any commercial or financial relationships that could be construed as a potential conflict of interest. The handling editor RJKV declared a past co-authorship with the author UA.

## Publisher's Note

All claims expressed in this article are solely those of the authors and do not necessarily represent those of their affiliated organizations, or those of the publisher, the editors and the reviewers. Any product that may be evaluated in this article, or claim that may be made by its manufacturer, is not guaranteed or endorsed by the publisher.
